# Ask the Doctor: Mental Hygiene Among the Young in Fin-de-Siècle Finland

**DOI:** 10.1093/shm/hkac039

**Published:** 2022-08-27

**Authors:** Tuomas Laine-Frigren

**Affiliations:** Postdoctoral Research Fellow, Faculty of Social Sciences (SOC), Academy of Finland Centre of Excellence in the History of Experiences, Tampere University, Tampere, Finland

**Keywords:** mental hygiene, young people, patient letters, nervousness, social medicine, Fin-de-Siècle, Finland, Konrad ReijoWaara

## Abstract

The turn of the twentieth century in Finland saw an increasing number of popular articles and books on health, which were published within the broader framework of ‘social hygiene’ and aimed at children, young people and their families. This article examines how young people articulated concerns about their own mental health in the context of these campaigns to improve social hygiene. Based on an extensive body of original sources consisting of medical advisory material and letters written by the young, the study reveals how young people saw themselves in this health context—especially when writing about their ‘nerves’ or ‘nervousness’. Drawing on more recent methodological investigations in the history of childhood, this study adds the much-needed perspective of the young people themselves as subjects experiencing these problems, to counterbalance the otherwise exclusively expert discourses on the subject of mental hygiene.

How wonderful the air is! It envelops our whole being, immersing us in such a fine bath that we do not even notice it, even while it makes us so powerful. It wafts the scent of plants indoors, transports ships across the ocean, and brings the crisp sea breeze and mountain freshness into our cities. Air is the carrier of light, and it brings to our ears the sounds of those we love.^1^

In 1899, extracts from *The Use of Life—*a book written by the British anthropologist, polymath and liberal politician John Lubbock (1834–1913)—were published in *Lukutupa*, a Finnish literary journal for young readers. The book was a characteristic example of Victorian self-help literature,^2^ on how to succeed in life without losing out on any of its joys. According to Sir John, our primary aim as individuals is ‘to make the most and best of ourselves’.^3^ Not only did happiness mean being industrious and morally upstanding, but also taking questions of health and recreation seriously; if a ‘young man’ was to forget ‘how to play’, he would soon be rendered powerless by gloom.^4^

The turn of the twentieth century in Finland saw an increasing number of popular articles and books on health, which were published within the broader framework of ‘social hygiene’ and aimed at children, young people and their families. Social hygiene strove to prevent disease by focusing greater attention on living environments, social and moral reform, and improving one’s overall lifestyle. Health education, therefore, covered many areas of life from advice about breast-feeding and the care of small children to tips on healthy clothing, cleaning the house, good nutrition, clean water, maintaining a good work–life balance and sexual morality for teenagers.^5^

This article examines how young people themselves expressed concerns about their own mental health in the context of these campaigns to improve social hygiene. I will show how focusing on the holistic well-being of children in both body and mind was a substantial part of the Finnish social hygiene movement. I will then proceed by looking at a unique collection of as-yet unpublished letters (see below) that reveal how young people saw themselves in this health context—especially when writing about ‘nerves’ or ‘nervousness’ (*hermostuneisuus*)—which also corresponds to prevalent medico-cultural concerns about neurasthenia in this period. Previous research has shown that neurasthenia was a particularly common fin-de-siècle diagnosis applied by medical professionals to describe an array of physical and mental symptoms also to be found in children.^6^ My argument is that nerves and nervousness provided a culturally flexible framework that allowed laypeople—including the young—to elaborate on their own personal problems.

By the end of the nineteenth century, the cultural presence of psychiatry and the neurosciences had spread to the Nordic countries, and scientific concepts like hysteria and neurasthenia were increasingly being used to discuss mental problems in Finland. It was also a time in which childhood became a key topic through which the hopes and fears of modernising nations in Europe could be articulated in terms of the future health, versatility and resilience of their citizens.^7^ In her pioneering research on children with nervous problems in late-nineteenth-century Finland, Jutta Ahlbeck highlights four fundamental assumptions about the possible root causes of their affliction. Children’s nerves were affected by one or more of the following: (i) an inherited weak constitution;^8^ (ii) increasingly intensive schoolwork in the modern classroom; (iii) gender—some believed the reproductive system in girls made them both physically and mentally weaker than boys; and (iv) an unenlightened education at home.^9^ Even if children with ‘weak nerves’ were not being diagnosed with a serious form of mental illness, they were nevertheless seen as a risk to the future vitality of the nation, which although still part of the Russian Empire now had a growing national movement.^10^ In addition, the theory of ‘degeneration’ that was currently in vogue across the world made the relatively new idea that even small children could go insane^11^ seem a very real and frightening prospect.^12^

According to the prevailing medico-political discourse at the time, enlightened Finnish citizens were seen to be those who were morally autonomous, took good care of their health, and exercised physical and mental self-restraint for the common good of the fledgling nation. Interestingly, although the final decades of the nineteenth century saw the emergence of Finnish-language institutions (e.g. state schools), the medical profession remained predominantly Swedish (for historical reasons).^13^ So it was that the leading proponents of social hygiene were originally Swedish speaking even if they were nearly all ‘Fennoman’ health activists (who wanted to make Finnish the national language). They were not only motivated by the thought of social and moral regeneration, but also that this enlightenment—via health reforms, social medicine and nation-building—actually happen in Finnish.^14^ International models and points of reference were sought, primarily from Germany (e.g. Rudolf Virchow’s ideas) and Sweden; although Britain too provided some interesting role models in social medicine.^15^

A key figure in the Finnish hygiene movement was Dr Konrad ReijoWaara (Relander).^16^ As one of the younger generation of Fennomans, he was a provincial medical officer, health activist and long-time editor of the ‘The Finnish Health Care Journal’ (*Suomen Terveydenhoitolehti*, est. 1889)^17^—the first popular healthcare magazine published in Finnish. What makes ReijoWaara stand out from other medical professionals of this period is his wholehearted commitment to improving general awareness of health issues among all Finns, regardless of their social standing. For instance, he published an astonishing number of popular essays and articles on all kinds of health-related issues (roughly 50 per year, and over 1000 in his whole career). It is clear that while ReijoWaara’s conservative moral convictions certainly motivated him in his mission to help his fellow Christians, it also meant he would judge them rather sharply on the level of individual habits (e.g. with regard to sexual issues). However, in his reformist views and practices, ReijoWaara and like-minded colleagues reveal a key difference between Fennoman medical professionals and many of their Swedish-speaking medical establishment;^18^ while the latter tended to espouse racial hygiene theories, the former steered away from the negative implications of degeneration and eugenics, emphasising instead the need to strengthen the vital, healthy and rational traits in people.^19^

Many of ReijoWaara’s writings promoted children’s well-being by discussing what he and some of his colleagues perceived as widespread health concerns. In so doing, he took a stand in the more general discussion about nervousness and its causes, and I will refer to some of these discussions in the first part of this article. However, more importantly, I will use the individual letters (which he responded to in his journal column) as a source in themselves. These letters detailed the particular concerns of individual readers who, though usually rural poor, were literate^20^ and had written to ask his advice. In fact, between 1889 and 1916, he received roughly 2,000 such letters, the majority of which he answered in this column.^21^ In their letters, which ranged from brief questions to detailed accounts of health, correspondents wrote about all sorts of ailments—anything from tuberculosis, digestion problems, headaches and boils to a wide range of nervous issues.^22^ In many cases, the correspondents would also ask the doctor’s opinion about a traditional or newly invented treatment they had learned about, either from some publication or friends who had already tried it.^23^

While most of these letters were sent by adults writing about their own problems, many of the letters concerned children, and some were actually written by teenagers and children themselves. As I will show, the diagnoses and diseases described in these letters reveal that some of these young people had adopted contemporary medical or ‘hygienic’ concepts and were using them to observe and manage their own health in conjunction with existing folk remedies (which might also be termed ‘lay medical cultures’).^24^ Insofar as none of these letters were published, they also represent a really interesting body of source material to study also the more intimate concerns of their young authors—for instance, as an alternative channel for articulating problematic everyday issues.

Many studies have shown how a new kind of medical concern for children’s mental health and personal development emerged in Europe and the Anglophone world during the long nineteenth and early twentieth centuries.^25^ Newly publicly funded schools and child protection systems were the outward signs of both broader socio-economic changes for children,^26^ and cultural ones in attitudes towards them. The idea of childhood as a time of innocence, education and play, requiring special treatment,^27^ had now begun to filter beyond the upper and middle classes and into the poorer segments of society.^28^ The result was that children were now seen to have special rights which, combined with other socio-economic factors, affected their position in modernising societies. There are also an increasing number of historical studies dealing with childhood and nation-building before the First World War (WWI). They have focused on the different contexts and practices of turning children and young people into ‘fit’ citizens—both in national and colonial arenas. This wealth of research illustrates how, insofar as being a uniquely challenging stage of life, childhood has been considered an important time for character building and inculcating beliefs and values via an informal means of education.^29^ However, these studies have usually been based on sources produced solely by adults. Drawing on more recent methodological investigations in the history of childhood, which take into account the perspectives and ‘voices’ of children in the past,^30^ this study adds the much-needed perspective of the young people themselves as subjects experiencing these problems, to counterbalance otherwise exclusively expert discourses on the subject.

## Nervous Children

The life-giving potential of fresh air cited at the start of this article—while lovely in itself—paints a somewhat idyllic picture, suggesting life opportunities often far from the socio-economic realities of many poorer families, who in reality often lived in cramped, dark, unhygienic accommodation in rural peripheries.^31^ Indeed, the everyday life of most Finnish children was characterised by general poverty, a dearth of schools, long working days, disease, poor nutrition, and sometimes hunger.^32^ Nevertheless, Lubbock’s lyrical portrayal is in tune with the idealism characterising Finnish health campaigns at this time. The idea was that if people collectively ‘woke up’^33^ and realised that it was in their best interests to cultivate body and mind, then this would greatly improve matters.

For the proponents of the hygiene movement, the existence of nervous children in society testified there was a need to improve the lifestyle and general social conditions of children, especially regarding prevailing practices in their education and nurture. In writing about children’s nervousness, they argued that a well-functioning nervous system—now seen as holistically governing both physical and mental well-being—relied on more than simply taking care of purely physical needs.

In January 1907, Dr ReijoWaara published a story about a girl called Maiju who was now 14 and had recently become troubled.^34^ Until then, she had been a perfectly happy, ‘rosy-cheeked’ child, but suddenly everything was different: she became ‘capricious’ and difficult to talk to—one moment closed in on herself, the next ‘bursting into nervous laughter’. She did badly at school, and even started treating her beloved cat harshly. She also confessed to her friend that ‘stupid things’ came into her mind she was ashamed of (such as wishing her family dead), and then one winter she really started to ‘sink into a state of deep sadness’. She lost her appetite, slept restlessly, became pale and felt a ‘heaviness’ in the head—especially after maths lessons. Faced with these strange changes, her mother was becoming increasingly anxious, so they went to see a doctor together. After meeting and examining her, the doctor explained that Maiju was going through the delicate ‘transition phase’ of turning into a woman. This was making it difficult for her to take the ‘nervous strain’ of school, so it was advised she take some time off school and be sent to the countryside where she could ‘ski, drink milk, and take part in the daily chores at the farm’. By the autumn, ‘Maiju returned to school blooming like a rose, in full health and with a renewed power to resist her earlier bouts of hysteria’.^35^

This little fable is characteristic of Dr ReijoWaara’s advice to parents (usually the mother) on how to address the everyday health issues of their children. Indeed, medical professionals in many countries during the late nineteenth century were arguing that an excessive focus on intellectual work would stymy children’s moral education and suppress the ‘vital energy’ necessary for healthy physical and mental development.^36^ In Finland, a mostly rural country, neurasthenia or ‘nervous weakness’ was also seen to be a working-class illness.^37^ It was also very much a socially prejudiced and gendered construct, with the chief risk group seen as girls and working-class children. Maiju’s story seems to be no exception—the gendered moral in such educational narratives^38^ was that girls were more fragile than boys. In this respect, it was her mother’s duty to take Maiju temporarily out of school to prevent her from growing into a woman who might well become ‘nervous, hysterical and weak’.^39^

Maiju’s story also reveals something about common perceptions of mental hygiene in the late nineteenth century. In these less serious forms of mental illness, a careful balance between work and rest was the answer plus plenty of outdoor rural activity. While it was culturally very common to consider children’s work in the countryside in moral and educational terms, it was also seen as healthy activity—not to mention plain useful in an overwhelmingly agrarian society.^40^ It was also a widely shared cultural norm among (especially rural) working-class parents that children mucked in with supporting the family economically;^41^ so throughout the nineteenth century (and in some cases beyond)^42^, work was often valued above school. These ideas and sociocultural norms were also manifest in public debates on children’s work at this time—they hardly ever dwell on rural child labour, focusing instead on industrial contexts.^43^

Nevertheless, especially those hygienists who had witnessed local practices first hand were critical of children they saw being ruined by excessive work.^44^ Health was of vital value—it was *life*^45^ itself—and to ruin it was simply wrong. As the journalist Vihtori Peltonen put it in 1894, ‘even the smallest [bodily or mental] disorder’ betrayed that a person was unwell, and we should bear in mind that the ‘spark of life was a precious gift, a sacred fire entrusted to our care’.^46^ It was the responsibility of every Finn to be proactive in promoting their (and the nation’s) health.

For ReijoWaara, work had the potential to heal in a number of ways. As well as its moral value for socialising young people into the national community, physical exercise had a concrete regenerative effect.^47^ According to contemporary neurology, by irritating the sick nerves, physical work stimulated a certain vital force in the body which, like a dynamo, had the positive knock-on effect of invigorating the will.^48^ Moderate amounts of work—especially outdoors and dressed in the proper clothing—steeled children for the rigours of life.

Finnish medical debates about ‘brain-forcing’ in schools resembled those going on in Britain at this time.^49^ At the turn of the twentieth century, many Finnish physicians and educators believed that school posed a potential threat to children’s nerves—especially when poorly organised. Some doctors believed it was the stress caused by a curriculum too focused on theoretical work,^50^ while others thought wider concepts of social hygiene should apply. One doctor, for instance, maintained that grammar school pupils were not ‘young slaves’ to be dulled by excessive work, but people who were in the delicate process of growing up. They would also need ‘freedom, fresh air, light, and love’ if they were to become people endowed with a ‘full imagination’ and mature emotional lives.^51^

Dr ReijoWaara expressed his opinion on the issue of brain forcing in 1896.^52^ He seemed to accept the popular medical claim that schoolchildren, ‘who had once been brisk, powerful and full of life’, were now often ‘nervous and weak’, despite the fact that many schools had actually been modernised with proper windows, ventilation and so on. But rather than blaming the school itself, he focused on what he saw as the weak resilience of children. While he admitted it was good that schoolteachers had flagged up the issue of ‘overburdened children’ at their national conference in Helsinki (1890), he disagreed with their proposed solution. Rather than hiring more in-school doctors, which he felt was too costly for such a small nation, ReijoWaara argued it would be better to provide families with proper information and advice. This would improve lifestyles and child-rearing practices which, by increasing the overall vitality and resilience of children, would lessen the likelihood of overburdening happening in the first place. Teachers should not worry about being too demanding, he argued, as without high standards children ‘would not survive on the battlefields of life’. As the ‘competition was intensifying in all sectors of life’, it was vitally important to make the children stronger in every respect.^53^ At the same time, he never got tired stressing the significance of rest—citing, for example, that the day of rest (*lepopäivä*) on the Sabbath was a great ‘treasure’ from which the nation had drawn its strength in the past and which would help its citizens in future to avoid ‘nervous weakness’. In the same vein, the young also had a right to be free from such hardships over the weekend, and so no homework should be given to them then.^54^

The notion of ‘weak nerves’ meant children with behavioural issues (i.e. a problematic ‘constitution’) to become focus of attention. In 1916, Dr Armas Ruotsalainen (1877–1958)^55^ started a discussion about mental illness in children by pointing out that signs of nervousness in children were ‘as diverse and manifold as life itself’.^56^ One special group, however, demanded special attention—tantrum children—who would ‘throw themselves on the floor’, reacting sometimes violently to parents as if ‘insane’.^57^ While sharing the commonly held view that the most likely reason for this behaviour was an ‘inherited disposition’,^58^ Ruotsalainen reminded parents that because they were more fragile to begin with, these children should be handled with great sensitivity. For the socially withdrawn, he suggested they be given useful and practical tasks to help around the house or garden and take up crafts or hobbies (like collecting plants) to support their personal growth and stimulate their interest in nature and life around them. The aim was ‘to show them that life is fun after all’.^59^

However, in serious cases of severe introversion, strange behaviour or deep depression, it was generally acknowledged that the ‘natural’ healing powers of work, the great outdoors or bathing in cold water might not be enough.^60^ In January 1916, for instance, one worried parent informed ReijoWaara that, unlike other children, her daughter ‘refused to go sledging’—even when ‘whipped’.^61^ In his answer, ReijoWaara skipped over the question of corporal punishment, focusing instead on the fact that if she was otherwise a ‘generally healthy girl’ who kept ‘bursting into tears for unnatural reasons’, then she should be taken to a ‘nerve specialist’ before her ‘nervous system’ became ‘ruined for good’.^62^ In another case, a boy from a relatively well-off family had been repeatedly stealing sweets and chocolate, and since whipping and ‘praying together’ had not helped at all, his mother had started wondering if there was something seriously wrong with her ‘childish 7-year-old’. In a written response in his column, ReijoWaara said that the boy might indeed have a mental disturbance known as ‘kleptomania’ and that he should be treated for it away from home.^63^

Many professionals advised that children be treated patiently, gently and without unnecessary punishment. Dr Ruotsalainen paid special attention to children with ‘weaker’ constitutions and stressed that they should not be frightened with punishments, threats or even ‘ghost stories’ for that matter.^64^ In an issue from 1908, Dr Onni Granholm had pointed out that children in their early years were ‘very sensitive to mental impressions’.^65^ Not only were they influenced by those who held them, but also by those who ‘spoke to them and looked at them’. In spite of not necessarily understanding the meaning of words, they could pick up on the non-verbal gestures of those looking after them—the warmth in the ‘tone of voice, the happiness or anxiety in their eyes, the softness or fierceness of their touch’.^66^ As one anonymous writer informed parents in a popular educational magazine from 1900, ‘the mind of a child is like soft wax’. If a child was not loved, ‘its mind would become inured to any positive influences.’^67^

While some popular educators were already recommending that all physical punishment be abandoned at the beginning of the twentieth century, in reality, it remained widely practised at both home and school.^68^ In folk culture, corporal punishment had a religious and magical function as well as a practical one: even 1-year-old infants were sometimes whipped to purify them from evil or the ‘original sin’, but hitting and slapping were also sometimes used as simply a reminder—sometimes even just to ensure a child was moving in the right direction, for example. However, not all rural working-class families used corporal punishment in the nineteenth century—as ethnologist Markku Aukia has pointed out—many folk traditions also discouraged unnecessary violence.^69^ The hygienists, too, generally believed that such punishment would only have the negative effect of driving children away, so the solutions they suggested were more positive such as, for instance, organising useful activities within the family.^70^ Even so, physical punishment was an integral part of a child’s everyday life, as indeed was the age-old practice of intimidating and frightening them—not only with the threat of punishment, but also with stories of fearful animals, supernatural monsters and sinister people.^71^ The proponents of children’s mental hygiene argued that this deeply rooted tradition of intimidating children with ‘elves, trolls or horned devils’ would have serious consequences for children’s nerves, and might even trigger a range of actual ‘nervous diseases’, such as epilepsy, hysteria or neurasthenia.^72^

In ReijoWaara’s correspondence, some parents suggested that their children had changed after experiencing great fear. For instance, in one letter, the mother complained that their 3-year-old boy had been frightened by the sudden attack of a dog and after this lost his ability to speak. In other cases, such as that of another 3-year-old who ‘saw mice and rats everywhere’, ReijoWaara would interpret the parents’ stories as proof that they needed to change their practices—in this case, he suggested that the child’s overly excited imagination was a consequence of being told too many exciting stories.^73^

Schools were also warned about how easily feelings of fear and anxiety could scar the developing minds of impressionable children, and so the intimidation of punishments and excessive discipline should be avoided at all costs. In one such article, for instance, published in the *Finnish Health Care Journal* in 1895, more than a dozen international experts^74^ were quoted, with accompanying statistics, as saying that school was far too mentally stressful. It showed how pupils in class very often had to put up with the ‘fear of punishment’, which not only damaged their ability to learn, but also their fragile minds. The author then went on to note that a nervous pathology called ‘*chorea minor*’, involving spasms, twitches and other involuntary movements, was particularly characteristic among ‘weak-blooded’ girls.^75^

Referring to Dutch sources, Nelleke Bakker has identified the process by which children’s difficult behaviour—traditionally managed by moral lessons, physical punishment and tales like Heinrich Hoffman’s *Struwwelpeter* (1845)—eventually came to be seen as a medical problem in need of special professional attention. As a time-bound cultural construct, ‘neurasthenia’ played an important role in this process. It referred to a phenomenon not yet fully medicalised, which was still tied to turn-of-the-century romantic and hygienic concepts of childhood.^76^ In light of the Finnish sources presented here, Bakker is certainly right in suggesting nervousness described *many* problematic, worrying and strange behaviours—disobedience being just one of them. At the same time, this implies that a comprehensive study of nervousness in children could really benefit by going *beyond* the limited perspective of medical expertise and include wider sociocultural and political concerns. While medical experts generally explained the problem in terms of ‘inherited disposition’, the reform-minded campaigners of social medicine were concluding that prevention, correction and reform were the best courses of action. As I will show in the final part of this article, the vocabulary of ‘weak nerves’ was also used by these young people as a means for self-observation, and as part of their everyday efforts to cope with their personal health issues.

## Long Letters and Self-diagnoses

As a provincial medical officer for three different localities in Finland, Dr ReijoWaara certainly came face to face with what many people were living through in their daily lives. Yet many of these problems, ranging from relatively minor health issues to more serious concerns (e.g. tuberculosis) and the aforementioned ‘nervous’ behaviour, came to his attention through the letters he received.^77^ We do not actually know the reason why ReijoWaara decided to start this practice of consultation via correspondence. Besides the possible educational motive of encouraging health literacy, he might also have considered the letters the best channel for receiving information from people about their state of health, behaviour and lifestyle for his grander overall project of wanting to help Finnish society as a whole become stronger. As a dedicated Fennoman physician and activist for social medicine, and like his medical counterparts in nascent Germany half a century earlier, who were equally motivated to improve the common lot,^78^ it was an excellent means for him to find out as much as possible about peoples’ everyday lives. Already, as a provincial medical officer, he had made arduous journeys to remote villages not just to cure people, but to also gather information on the general health of the population in certain areas.^79^ For instance, there was a study he made on the health and illness of the rural population in one of his districts that went into comprehensive socio-economic detail.^80^ His correspondence could, therefore, be seen as some kind of qualitative extension of his research activities.

Anssi Halmesvirta has suggested that ReijoWaara might well have considered himself a kind of ‘agony uncle’—continuing a much older tradition, but in the rejuvenated context of the Finnish national romantic movement. Perhaps he could also have seen himself as providing some kind of remote ‘therapy’ where individuals could bear their hearts in a way that was not possible at home. In this section, I will start from this premise and examine more closely how some of his correspondents expressed their intimate problems in writing. After that, I will hone in on one particularly interesting and detailed letter written by a 15-year-old girl.

Many of these letters concerned seemingly mundane issues, such as pimples, warts, freckles or the loss of hair,^81^ but they were often also driven by genuine worry. One 16-year-old boy, for example, started by recounting that he had found a barely perceptible ‘hole’ in his neck, and asked ReijoWaara why there was some kind of strange ‘fluid’ flowing out of it. The boy also mentioned that he suffered from a bad cough and got tired easily, but he did not believe it was tuberculosis, as his ‘chest wasn’t aching’ and he had a ‘great appetite’. Perhaps the cough was making the liquid come out, he wondered, and it might eventually be for the good.^82^ Looking beyond the content of the letter, it is tempting to ask why he felt the need to write the letter, if he really thought it was nothing serious. Perhaps nobody wanted to listen to him at home, and this is one example of letters being used as an alternative channel for articulating problematic everyday issues.

Quite a few of the letters came from adolescents whose ailments were in one way or other related to sexual anxieties. Very often the reason for these confessional letters was ‘onanism’ or ‘self-pollution’ (*itsesaastutus*), as masturbation was then known. Masturbation was considered an outward sign of neurasthenia and, in a culture still dominated by the Lutheran church, it was seen as both bad for the health *and* immoral.^83^ Little wonder, therefore, that the teenagers who confessed these habits to the doctor seemed rather depressed—expressing feelings ranging from shame (related to their future ability as spouses) to guilt, to fears of going blind, going crazy or dying.^84^ One reason for their fears seems to have been the way they holistically interpreted all kinds of ailments and symptoms they felt to be connected to masturbation. One ‘16¾-year-old’ boy, for instance, was very concerned about his chronic headaches, the weird feelings in his chest, and aches in his hands and feet. He confessed that he was also nervous, ‘slept restlessly’, ‘dreamt a lot’ and ‘got easily frightened’. He asked if all these might be due to the practice of ‘onanism’ he had fallen prey to for the last 2.5 years.^85^ As well as the inherent religious sensibilities here, these adolescent fears were aggravated by theories circulating in fin-de-siècle medicine that masturbation could result in a very concrete loss of life force and ability to reproduce (due to the outflow of ‘seed’),^86^ and this clearly had an impact on these teenagers’ emotional lives.^87^

While the young letter writers often did refer to ‘onanism’ in their self-diagnoses, this might have again been simply a pretext for writing to the doctor about other issues related to health. The following three cases illustrate this. In the first, a 16-year-old shop assistant connected his feelings of insecurity to having once been an onanist, describing how ‘nerve-wracking’ it was to socialise with the customers’ everyday feeling that people could ‘see through’ him. The job was not helping him recover, and he was convinced his feelings of insecurity would never go away.^88^ The second started by recounting how ‘restlessness’ and ‘deep melancholia’ were causing the letter writer to fear for his very sanity. He put this down to having formerly been an onanist, but then went on to complain about unhealthy conditions at the factory he worked in, adding that he was now ‘an eager friend of fresh air’ and was heading ‘to the sea’ as soon as his health would allow it.^89^ In the last case, a depressed 18-year-old boy from Helsinki complained about how his head was ‘sensitive to being touched’. Again, he started by connecting this initial problem to having been an onanist earlier, but then went on to describe more pressing problems with his peers. Not only had they been the ones that had originally ‘taught’ him these things, but they were continuing to show off their ‘manliness’ by talking in lewd terms, so he felt it would be best for his health ‘to walk alone’. The boy also recounted how he had acquainted himself with the self-help manuals by ‘Dr Sylvanus Stall’^90^ and had tried to ‘cram as much information into his head as possible’, and that he had been skiing, skating and doing exercises at home to improve his health and keep himself on the right track. None of this had helped though, so he was asking ReijoWaara for ‘fatherly advice’.^91^

ReijoWaara’s responses to many of these letters were typically quite straightforward, and as such it might have been a relief for some readers as they could carry out these ‘cures’ themselves at home. He reassured one 17-year-old, for instance, that there was no reason to lose hope as he had clearly recognised his flaws by admitting that others had lured him into practising onanism. From now on, it would be enough to follow a healthy way of life^92^ to restore his vital energies. While it seems clear that the content of these answers would hardly offer satisfying solutions for many of the problems these teenagers were facing, the act of correspondence might have had a positive effect in and of itself, by allowing the correspondents to express awkward issues they might otherwise have not been able to get off their chests.

Boys were not the only ones to write to the doctor: in October 1914, an 18-year-old schoolgirl^93^ started her letter as an almost religious confession of guilt which then evolved into a story of failed romance, uncontrollable ‘dreaming’, melancholy and nervous exhaustion. She explained that she had already tried talking about it to a ‘Reverend Kares’, but now it was the doctor’s turn to tell her what to do. At the age of 10 or 11, she had read a book called *What a Young Girl Ought to Know*,^94^ and only then realised that what she had been doing with herself was bad. After that, a long time had passed happily, but then the ‘temptation’ had come back when half asleep at night and in the form of dreams. Looking back at that period in her life, she had found a reasonable explanation for these ‘intense struggles’, as she was in the process of choosing a ‘mission in life’—quite possibly, this mission was religious.

However, things had recently started to get worse because she had met someone. At first, the love had been ‘pure’, but then her ‘friend’^95^ had ‘embraced’ her in ways that caused her former sin to ‘haunt’ her again. Before long, she was having trouble sleeping, finding that at 1 am every night she was ‘lapsing’, after which she would spend the rest of the night weeping with her nerves ‘ruined’. However, in an interesting change of emotional register at the end of the letter, she notes that ‘temptation’ was no longer the real issue anymore^96^ as the friend had left her so that her passion seemed to have ‘burned out in the grip of pain’ and now she was only sad. Because of all these emotions and the lack of sleep, her ‘nerves’ were now overwhelmed: she could hardly work, study or even think because of the ‘pressure in her head’. In a surprising twist at the end, she then wonders if the doctor could write a letter explaining her situation and his advice on what to do to Reverend Kares on her behalf. As in other cases mentioned above, the vocabulary of ‘weak nerves’ once more provided this person with a vehicle to work through her feelings of loss and hopelessness in a constructive way.

Not all these letters dealt with sexual issues. In March 1909, a girl aged between 15 and 16 from the village of Inha in Southern Ostrobothnia wrote a long letter to ReijoWaara (nearly 16 pages of small handwriting), describing mainly how she had been suffering from chronic pains in the stomach, but also mentioning emotional issues and interesting details about her own efforts to cope with bad health. She seemed to know quite a lot about contemporary health campaigns (having read ReijoWaara’s journal, for instance) and makes many self-observations.^97^ In what follows, I am particularly interested in how she diagnoses herself.

As she came from a crofter family,^98^ she had to work for the landlord—like many working-class girls in the countryside—doing jobs like housework, baby minding and herding animals. Crofter farms were usually run jointly by families working together, and children’s contribution to the household economy was often vital.^99^ Like most Finns, she lived in the country but, as crofters, her family’s social position was probably more secure than it was for a family of day labourers (the majority of the rural population). In these families, the children would have to accept almost any work offered due to the household’s extremely insecure daily income. Many tenant farmers, however, did also face this struggle for subsistence—a poor selection of food, bad nutrition, disease and hunger.^100^ At the same time, the fact that this correspondent wrote so well proves that she had spent at least a few years in school.

At the start of the letter, she describes what she terms ‘gastritis’, and wants to know the best diet for her to overcome this. She has apparently always suffered from ‘bad digestion’ and suspects that this is because of all the ‘horrible food’ she has been forced to eat over the years: both from the landlord—‘very salty, barely cooked and hardly ever heated’—and from her parents. The complaint levelled at her parents particularly highlights their superstitious beliefs: ‘[…] when I was small I suffered from cramps and spasms for 6 months, and all kinds of quack remedies were used by my parents to fight the disease. […] I had to consume bear bile, frog’s blood, wine, and other kinds of stuff like that, so it’s only natural the gut might suffer after that […]’. But food was generally scarce anyway, and she wrote that there were many winters when she ate mostly food that was not even cooked, like ‘cowberry jam mixed with rye flour’.

By mentioning the poverty of her family so many times, it seems as if she was convinced that the poverty itself was the reason why there was no proper information about questions of health and hygiene in her family, or at least a general disregard for them. This was the main reason, she believed, as to why she had not yet received proper advice about the best way to treat herself. In some ways, she seems ashamed of her family in her long letter: ‘they don’t really care about their health’, she explained, going on to complain about how the rest of the family sometimes bullied her and made fun of her for being precocious and too proud, in spite of the fact, she added, that ‘I’ve only been trying to look after myself.’ Her mother also belittled these efforts by saying to her ‘you think you’re better than the rest of us’, and then adding that there was no point trying to improve matters, as she was ‘simply smaller and weaker than all the other children’. The girl explained in the letter that she had decided to stop eating potatoes or drinking coffee for 4 weeks in a row—something which must have been very hard for such a family. However, because nobody took her troubles seriously she had kept silent about her pains, and she confessed to the doctor that this was actually the first time she had ever told anyone about them. In writing to a middle-class doctor, this rural working-class author was perhaps looking for reassurances about the kind of person she was hoping to become. As such, this could be seen an epistolary act of identity building^101^ within the framework of health and hygiene.

She starts the letter with a detailed depiction of her stomach problems, then the focus suddenly switches to an experience she had 4 years before. She was doing her homework outside with her brother (reading the Bible), when she suddenly felt a strange feeling ‘inside her head’ and then felt unable to see or hear anyone around her. Becoming very frightened, she went to lie down on her bed. The next day at school she had a similar attack which lasted several hours, but she was not as afraid as she had been the first time and so acted as if ‘nothing had happened’. Instead, she tried to be as ‘happy as the rest of the children’. Interestingly, she had her own explanation for these strange sensations—recently an old woman that she and the other children knew had just died. As the woman lived nearby, they decided to go ‘see the body’ and, according to the girl, ‘it was the first time I had seen a dead human being.’ The following night, when she found she could not get to sleep, the girl found herself lying awake ‘thinking of the dead woman’, feeling like the corpse was ‘lying on the floor’ next to her in the room.

That summer, the weird ‘dizziness’ came back, and again the girl seemed convinced that it had something to do with death—another local woman had suddenly died, this time ‘while making coffee’. The girl had just gone to bed when her brother rushed into the house to ‘announce’ that ‘the grim reaper had come a-knocking again’. She recounts in the letter ‘I got so scared that my body was trembling, my heart started pounding, I felt sick and my head hurt.’ After this, she began to have the dizzy spells again, sometimes mild, sometimes severe, and now, as she approached the age of 16, they were still with her. ‘Especially when I’m walking in the forest,’ she wrote, ‘there’s a fear I’ll get lost and a nasty feeling inside me all the time. Hardly anything makes me happy or brings me pleasure any more, not the beauty of spring, winter, or the autumn.’ And these feelings surfaced in other kinds of situations too, both when there were other people around and even when she was alone reading—everything suddenly started to look strange.

The letter ends on a rather depressing note; bearing in mind the mental troubles she just described, she believed she would always have these stomach problems, not to mention the fact that her muscles—especially in her arms—had become ‘thin and weak’. Not even work was giving her the same pleasure as it had previously—when she still had plenty of energy. Finally, she noted that she might well have a serious problem with her nerves. Her self-diagnosis was that she was in a state of shock caused by her encounters with death; this, in turn, had been physically and mentally exacerbated by her poverty. However, while she certainly did get tired easily, she remained hopeful—confessing that she could still summon the energy to do some work, such as ‘dragging firewood with a sledge’.

According to Turo-Kimmo Lehtonen, the Finnish social hygiene movement tended to see health as the *absence* of illness and disorder. As long as a person was healthy, there was a greater chance for happiness to prevail.^102^ For the girl who wrote the long letter, illness was clearly something that stopped her from being able to fully take part in the responsibilities of everyday life. As she saw it, she was ‘withering away’ and felt incapable of fulfilling the social expectations of a girl her age at that time. This was shown in how she described her condition in terms of those jobs she could no longer do, and the work she should perform to remedy her ‘digestion’ problems. In this respect, her account endorses prevailing cultural and medical views about the positive effects of work. Perhaps, the act of writing about the nervous and physical effects of poverty also provided an outlet for the letter’s author to work out her own changing position in the family, as she was growing up and learning about new things that were difficult for her to explain to them otherwise. In a way, this letter can also be seen as answering the call, albeit unwittingly, of health campaigners who argued that individuals would best reach personal and political maturity via an enlightened awareness of their own health.

## Asking or Telling the Doctor?

The story of ‘mental hygiene’ usually begins in the early twentieth century—or in the more specific context of children—with the first child guidance clinics set up in the USA and child psychoanalysis in Europe after WWI.^103^ Indeed, the first child guidance clinics in Finland (and public health campaigns specifically targeted at children’s health) were associated with social organisations founded during the interwar period, such as *General Mannerheim’s League for Child Welfare* (est. 1920) and *Folkhälsan* (est. 1921), which both aimed to improve the quality of youth in the country.^104^ New psychological discourses and practices also began to be deployed in tackling mental instability in children and tracing the root causes of criminality and deviance.^105^

This article complements a psychology-centred narrative by placing a doctor’s advice^106^ in the wider context of social medicine at the turn of the twentieth century. I have paid special attention to Dr Konrad ReijoWaara’s active agency in advising people. As shown by Anssi Halmesvirta, ReijoWaara’s ‘consultation by correspondence’ was a rather unorthodox method for treating patients at this time in Finland. It was not an examining gaze that investigated the body and mind from an exclusively medical perspective, but an ‘open means of listening to the desperate voices of the afflicted’.^107^ Maybe the rich variety of ideas and feelings that he encountered in the letters created a snowball effect which then inspired him to pursue his wider project of increasing the overall number of health-conscious Finnish citizens.

It was also significant that ReijoWaara, unlike many fellow Swedish-speaking colleagues, communicated with his readers in Finnish, which made him easier to reach for most of the population. Rather than prescribe medicine, most of his advice encouraged do-it-yourself cures involving bodily hygiene, a good work–life balance, gymnastics, fresh air, sunlight and healthy activities in a natural outdoor environment. In this respect, his outlook as a hygienist was social and environmental,^108^ and it rejected the theories of eugenics that were becoming increasingly fashionable at that time. Preventing mental illness by tapping into the natural *élan vital* of children was considered by ReijoWaara and like-minded colleagues to be of the utmost importance in building the modern nation-state.

This study has shown how an active medical interest in the mental well-being of young people developed within the framework of an overall movement for improving the general health of the Finnish population at the turn of the twentieth century. In particular, the culturally influential discourse on ‘nervousness’ and ‘weak nerves’ acted as a catalyst for professionals to discuss mental issues in a wider context. The discourse surrounding nervous problems at this time did not place the mental and somatic spheres in two different categories as it would do later on; rather, it conceptualised them as an indivisible whole, and combined them with normative, hygienic and romantic notions of a healthy childhood. By following international models and adapting them to the Finnish context, preventive social and mental hygiene aimed to conceptualise children’s needs decades before the post-WWI mental hygiene movement. These ideals of social medicine and research in Finland remained popular until the divisive Civil War (1918), after which the earlier optimism attached to medical social research withered away.^109^

Perhaps the most important way this article complements a psychology-centred narrative is by shifting the focus away from expert (and adult) perspectives on mental hygiene to those of the young people themselves. Through a unique collection of letters, I have looked at how they personally viewed and dealt with their ‘nervousness’. In these letters, teenagers are inquiring about health problems and other intimate issues that had been bothering or frightening them. Very often, they were scared of what nowadays we would call masturbation but at the time was masked in complicated euphemistic terms such as ‘onania’ or ‘self-pollution’—a sad sign of how prevailing medico-moralist conceptions of sexuality may only have exacerbated young people’s anxiety. Interestingly enough, the terminology of ‘nerves’ seemed to treat body and mind holistically as intertwining spheres, which would explain why the 18-year-old with the head that was ‘sensitive to being touched’ not only believed that this sensitivity was a physical symptom of his past masturbatory activities, but also why he really thought that reading as much as possible could physically stop such symptoms too.^110^ The process of writing to the doctor was also giving these teenagers an outlet to work through what was troubling them that was denied them at home.

Especially interesting were those letters that seemed to reveal something more about the personal challenges these young people were facing—such as the long letter featured in this article from the adolescent girl who related her condition to her family’s overall general poverty, total lack of medical education and nervous weakness. It is clear, as with the schoolgirl who had clearly experienced some form of sexual love for the first time, that we cannot know precisely what these letter writers were going through, but the quality of detail would suggest that we do not limit our research to only those experiences which we can verify as being truly authentic. Rather I propose that these kinds of letters (and other ‘ego documents’) offer exciting methodological possibilities for studying the fin-de-siècle discourse surrounding childhood, youth and ‘nervousness’. They are pertinent not only to the academic topic, but also as socially meaningful responses to real-life issues and crises.

It is for this reason I am asking exactly *why it is* that these teenagers were writing to a remote doctor using the vocabulary of contemporary academic medicine. My first answer would be that this form of correspondence offered a ‘safe’ way to deal with intimate health-related issues that could not be discussed at home. It was thus an alternative channel for information. Secondly, they were perhaps using medical ideas as a tool to resolve their own health issues and personal life situations—and this clearly seemed natural to them. This would suggest that these letters, and others like them, are worthy of further research as they demonstrate identity building within the wider framework of late nineteenth- early twentieth-century health and hygiene. By digging deeper into such contemporary sources, we might reveal more about how their medical notions correlated with (or indeed diverged from) prevailing cultural norms and expectations (e.g. gender, sexuality and nation-building), and how all these factors influenced their everyday lives. As such, the historical importance of these letters lies less in what advice they elicit from the doctor and more in what the children choose to reveal about themselves and their situation.

## Footnotes

1 Lukutupa. Kaunokirjallinen nuorisolehti 29 April 1899, 75.

2 See Eva Illouz, *Saving the Modern Soul: Therapy, Emotions, and the Culture of Self-help* (Berkeley, Los Angeles and London: University of California Press, 2008), 152–53; Tom Crook, ‘“Schools for the moral training of the people”: Public Baths, Liberalism and the Promotion of Cleanliness in Victorian Britain’, *European Review of History: Revue Européenne d’histoire*, 2006, 13, 21–47. Bruce Haley, *The Healthy Body and Victorian Culture* (Cambridge: Harvard University Press, 1978).

3 John Lubbock, *The Use of Life* (New York and London: Macmillan & Co, 1894), 5. https://archive.org/details/uselife02lubbgoog/page/n16/mode/2up.

4 Lukutupa. Kaunokirjallinen nuorisolehti 29 April 1899, 75.

5 Minna Harjula, *Hoitoonpääsyn hierarkiat* (Tampere: Tampere University Press, 2015), 41; Anna-Maria Mäki-Kuutti and Sinikka Torkkola, ‘Tarkkuuskello ja höyrykone: terveysoppaat sukupuoleen kasvattajina’, *Kasvatus & Aika*, 2014, 8, 37–53; Minna Harjula, ’Den hygieniska medborgaren. Hälsoupplysningens kris i Finland i början av 1900-talet’, in Johanna Annola, Annelie Drakman and Marie Ulväng, eds, *Med tvål, vatten och flit: Hälsofrämjande renlighet som ideal och praktik ca 1870–1930* (Lund: Nordic Academic Press 2021), 183–207.

6 There are only a few studies focusing on neurasthenic children: for example, Nelleke Bakker, ‘Before Ritalin: Children and Neurasthenia in the Netherlands’, *Paedagogica Historica*, 2010, 46, 383–401; and Jutta Ahlbeck, ‘The Nervous Child and the Disease of Modernity’, in Jutta Ahlbeck, Päivi Lappalainen, Kati Launis and Kirsi Tuohela, eds, *Childhood, Literature and Science. Fragile Subjects* (London and New York: Routledge, 2018), 170–83. Instead, they have been more commonly discussed in the wider context of medical and cultural modernity, for example, David Schuster, *Neurasthenic Nation: America’s Search for Health, Happiness, and Comfort, 1869–1920* (Piscataway: Rutgers University Press, 2011), 44, 60; and Volker Roelcke, ‘Electrified Nerves, Degenerated Bodies: Medical Discourses on Neurasthenia in Germany, circa 1880-1914’, in Marijke Gijswijt-Hofstra and Roy Porter, eds, *Cultures of Neurasthenia. From Beard to the First World War* (Leiden: Brill Clio Medica, 2001), 177–97. For a new take on neurasthenia as a historical precursor for modern notions of distress, see Petteri Pietikäinen and Jari Turunen, ‘Diagnoses In and Out of Time: Historical and Medical Perspectives on the Diagnoses of Distress’, *Diagnosis*, 2017, 4, 3–11. Meanwhile, in the Nordic context, there is Minna Uimonen, *Hermostumisen aikakausi. Neuroosit 1800–1900-lukujen vaihteen suomalaisessa lääketieteessä* (Helsinki: SHS, 1999); and Petteri Pietikäinen, *Neurosis and Modernity. The Age of Nervousness in Sweden* (Leiden: Brill, 1998).

7 Sally Shuttleworth, *The Mind of the Child. Child Development in Literature, Science, and Medicine, 1830-1900* (Oxford: Oxford University Press, 2010).

8 Cf. Siân Pooley, ‘“All We Parents Want Is That Our Children’s Health and Lives Should Be Regarded”: Child Health and Parental Concern in England, c.1860–1910’, *Social History of Medicine*, 2010, 23, 528–48.

9 Ahlbeck, ‘The Nervous Child’, 173.

10 The problems of mental health were becoming topical anyway as the number of mentally ill seemed to be increasing. The number of insane was thought to be alarming also in Sweden, see Pietikäinen, *Neurosis and Modernity,* 34.

11 See, for example, Steven J. Taylor, ‘“She Was Frightened While Pregnant By a Monkey at the Zoo”: Constructing the Mentally-Imperfect Child in Nineteenth-Century England’, *Social History of Medicine*, 2017, 30, 748–66.

12 For an extensive contemporary Finnish study on children’s hysteria, see Arthur Clopatt, *Etudes sur l’Hysterie infantile* (1888). For more on French discussions about this (and the influence of Clopatt’s work) in the same period, see Mark S. Micale, *Hysterical Men. The Hidden History of Male Nervous Illness* (Cambridge: Harvard University Press, 2008), 183–87.

13 Tuomas Laine-Frigren, Markku Hokkanen and Jari Eilola Eilola, ‘Sufferers, Specialists, Spaces and Society’, in Tuomas Laine-Frigren, Jari Eilola and Markku Hokkanen, eds, *Encountering Crises of the Mind: Madness, Culture and Society, 1200s-1900s*, (Leiden: Brill, 2018), 21.

14 On the rise of cultural and political Fennomania and the Finnish nation-building, see Jason Lavery, *The History of Finland* (Westport, Connecticut and London: Greenwood Press, 2006), 51–70. On Fennoman ideas of social engineering, see Petteri Pietikäinen, *Kipeät sielut. Hulluuden historia Suomessa* (Helsinki: Gaudeamus, 2020), 42–46.

15 The English medical establishment’s reaction to the cholera epidemic of the 1830s was particularly admired in Finland as a historical starting point for modern public health work. Harjula, *Hoitoonpääsyn hierarkiat,* 43

16 In 1906, Konrad Relander (1853–1936), a dedicated Fennoman, decided to finnicise his name (from Swedish) to ‘ReijoWaara’, just like tens of thousands of other people did in that year to celebrate the centenary of the ‘national philosopher’ and statesman Johan Vilhelm Snellman.

17 Pertti Mustajoki, *Mies joka hakkasi halkoja. Terveysapostoli Konrad ReijoWaara* (Helsinki: Duodecim, 2013), 125–29.

18 In fact, Swedish-speaking Finns had their own popular health care journal, *Tidskrift för Hälsovård* (1891–1918), edited by Wilhelm Sucksdorf.

19 Anssi Halmesvirta, *Vaivojensa vangit. Kansa kysyi, lääkärit vastasivat - historiallinen vuoropuhelu 1889–1916* (Jyväskylä: Atena, 1998), 16–17.

20 In Finland, functional literacy (i.e. reading and writing skills for real-life purposes) had significantly increased during the latter part of the nineteenth century. See Laura Stark, ‘The Rise of Finnish-Language Popular Literacy Viewed Through Correspondence to Newspapers 1856-1870’, in A. Edlund, L. Edlund and S. Haugen, eds, *Vernacular Literacies—Past, Present and Future* (Northern Studies Monographs 3. Umeå: Umeå Universitet, 2014).

21 These letters unexpectedly came to light in the mid-1990s, when the historian, Anssi Halmesvirta, came across them when going through the archives of *Duodecim* (the Finnish Medical Society). Dr. Halmesvirta salvaged the letters from boxes under layers of dust in an attic, photocopied them and handed these copies to me upon his retirement. The original letters remain in the *Duodecim* archives in Helsinki. For previous studies focusing on these letters, see, for example, Anssi Halmesvirta, ‘Despair in Finnish: Consultation by Correspondence in Fin-de-Siècle Finland’, in Laine-Frigren, Eilola and Hokkanen, eds, *Encountering Crises of the Mind*, 139–62.

22 In September 1913, for example, a 36-year-old village-school teacher sought help from Dr ReijoWaara in which she wrote that she was losing the love she had previously always had for teaching and the children. Although she was now lucky enough to finally have a classroom of her own—with ‘windows and all’—she had been having ‘severe bouts of crying’, and now she felt she had simply ‘lost the joy of life’. The teacher confessed that over the years, she had not really been taking proper care of herself, although last summer she had been lucky to spend a week in the Turku archipelago, where she had ‘swum in the ocean every day’, and felt the positive and relaxing effects of the ‘sea breeze’. She had also tried out what she called ‘J.P. Müller’s gymnastic system’. In his column, ReijoWaara answered back by saying that she was clearly suffering from ‘neurasthenia’ and reassured her that it would pass with time. However, if she did become depressed again, it might be a good idea to go and see a doctor in person to get her *elämänrohkeus*, or ‘courage to live’ back again. See Letter from Humppila to ReijoWaara, 19 September 1913, and *Suomen Terveydenhoitolehti* 10/1913, 168 ‘Opettaja’ (1497).

23 Very often, these were natural body management practices that were becoming increasingly trendy in early twentieth-century Finland. See Suvi Rytty, *Ruumiista reformiin. Suomalaiset elämänuudistajat, luonnonmuokainen ruumiinmuokkaus ja modernisaation ongelma, 1910–1932.* Dissertation (Turku: University of Turku, 2021). https://urn.fi/URN:ISBN:978-951-29-8681-1

24 Sonja Boon, *Telling the Flesh. Life Writing, Citizenship, and the Body in the Letters to Samuel Auguste Tissot* (London: McGill-Queen's University Press), 3–4. In previous research, letters and medical correspondence have been used to examine lay definitions of illness and health, but these studies usually concern earlier periods, and the ‘voices’ they reveal often belong to upper classes. See, for example, Willemijn Ruberg, ‘The Letter as Medicine: Studying Health and Illness in Dutch Daily Correspondence, 1770–1850’, *Social History of Medicine*, 2010, 23, 492–508; Wayne Wild, *Medicine-by-post: The Changing Voice of Illness in Eighteenth-Century British Consultation Letters and Literature* (Amsterdam and New York: Rodopi, 2006).

25 Shuttleworth, *The Mind of the Child*; Carolyn Steedman, *Strange Dislocations. Childhood and the Idea of Human Interiority 1780-1930* (London: Virago Press, 1995); Ahlbeck, ‘The Nervous Child’. For psychiatric constructions of children’s emotional lives in nineteenth- and twentieth-century Finland, see Mikko Myllykangas and Katariina Parhi, ‘The Unjustified Emotions: Child Suicide in Finnish Psychiatry from the 1930s Until the 1970s’, *Journal of the History of Childhood and Youth*, 2016, 9, 489–508.

26 For instance, technological developments and increasing productivity towards the end of the nineteenth century rendered child labour increasingly redundant. See Marjatta Rahikainen, ‘School, Child Labour and Rationalization in Industrialising Europe’, in G. K. Lieten and Elise Nerveen Meerkerk, eds, *Child Labour’s Global Past, 1600-2000* (Bern: Peter Lang, 2011), 97–117; Marjatta Rahikainen, ‘Lasten työnteko ja lapsityövoiman kysyntä’teollistuvassa Suomessa’, in Juhani Koponen and Sakari Saaritsa, eds, *Nälkämaasta hyvinvointivaltioksi. Suomi kehityksen kiinniottajana* (Helsinki: Gaudeamus, 2019), 151–66.

27 Steven Mintz, *Huck’s Raft. A History of American Childhood* (Cambridge and London: Harvard University Press, 2004). For the role of literature in changing notions of childhood in Nordic countries, see Kati Launis, ‘The Birth of the Modern Child in Finnish Literary Realism,’ in Ahlbeck, Lappalainen, Launis and Tuohela, eds, *Childhood, Literature and Science*, 60–72; and Olle Widhe, ‘Inventing Subjectivity and the Rights of the Child in Nineteenth-Century Nordic Children’s Literature’, in Reidar Aasgaard, Marcia J. Bunge and Merethe Roos, eds, *Nordic Childhoods 1700-1960. From Folk Beliefs to Pippi Longstocking* (London: Routledge, 2018), 265–82.

28 Steven J. Taylor, *Child Insanity in England, 1845-1907* (London: Palgrave Macmillan, 207), 2. Kaisa Vehkalahti, ‘Kasvatuslaitos modernin kansalaisuuden rakentajana,’ in Anja Heikkinen & Pirkko Leino-Kaukiainen, ed, *Valistus ja koulunpenkki. Kasvatus ja koulutus Suomessa 1860-luvulta 1960-luvulle* (Helsinki: SKS, 2011), 389–90. Cf. Clark Nardinelli, ‘Corporal Punishment and Children’s Wages in Nineteenth Century Britain’, *Explorations in Economic History*, 1982, 19, 283–95.

29 Hugh Morrison, ‘Settler Childhood, Protestant Christianity and Emotions in Colonial New Zealand, 1880s–1920s’, in Stephanie Olsen, ed, *Childhood, Youth and Emotions in Modern History. National, Colonial and Global Perspectives* (UK: Palgrave Macmillan, 2015), 76–94. For more on informal means of education and adolescent emotional loyalties in the context of British militarism, see Stephanie Olsen, *Juvenile Nation. Youth, Emotions, and the Making of the Modern British Citizen, 1880-1914* (London, New Delhi, New York and Sydney: Bloomsbury, 2014); Silvana Vilodre Goellner, Sebastião Josué Votre andMaria Claudia Brandão Pinheiro, ‘“Strong mothers make strong children”: Sports, Eugenics and Nationalism in Brazil at the Beginning of the Twentieth Century’, *Sport, Education and Society*, 2012, 17, 555–570; Halmesvirta, ‘“Building the Élan Vital”; Ivar Wilskman’s Ideology of Gymnastics’, *The International Journal of the History of Sport*, 2009, 26, 621–39. As regards ‘child exhibitions’ in early twentieth-century Finland, see Pirjo Markkola, *Työläiskodin synty. Tamperelaiset työläisperheet ja yhteiskunnallinen kysymys 1870-luvulta 1910-luvulle* (Helsinki: Suomen Historiallinen Seura), 193–95. See also John Neubauer, *The Fin-de-Siècle Cult of Adolescence* (New Haven and London: Yale University Press, 1991).

30 Nell Musgrove, Carla Pascoe and Kristine Moruzi, ‘Hearing Children’s Voices: Conceptual and Methodological Challenges’, Kristine Moruzi, Nell Musgrove and Carla Pascoe Leahy, eds, *Children’s Voices from the Past* (Palgrave, 2019), 1–5; Sian Pooley, ‘Child Philanthropy, Family Care and Young Bodies in Britain, 1876-1914’, Sian Pooley and Jonathan Taylor, eds, *Children’s Experiences of Welfare in Modern Britain* (London: University of London Press, 2021), 99–122. For a classic essay on the sociology of childhood, see Leena Alanen, ‘Rethinking Childhood’, *Acta Sociologica*, 1988, 31, 53–67.

31 Pirjo Markkola, ‘Negotiating Family, Education, and Labor. Working-Class children in Finland in the Nineteenth and Twentieth Centuries’, in Aasgaard, Bunge and Roos, eds, *Nordic Childhoods*, 163–174. In 1900, around 87 per cent of Finns lived in rural areas and agriculture was the main source of income for most of the Finnish population. However, less than a half of these people earned their living by independent farming.

32 Ibid., 169.

33 Konrad ReijoWaara, ‘Liittoon nuoret ja vanhat kansan terveyttä suojelemaan ja nostamaan’, *Terveydenhoitolehti*, 1906, 18, 97–101.

34 Konrad ReijoWaara, ‘Tytöstä naiseksi kehittyessä’, *Terveydenhoitolehti*, 1907, 19, 1.

35 ReijoWaara, ‘Tytöksi naiseksi kehittyessä’, 5.

36 Schuster, *Neurasthenic Nation*, 44.

37 See Hugo Holsti, ‘Heikkohermoisuudesta’, *Suomen Terveydenhoitolehti*, 1897, 5, 65–69. In his article on ‘nervous weakness’, Holsti stressed that nervousness occurred in men, women and children from *all* social classes. One particular concern among doctors was that the great emigration waves to the USA indirectly caused nervous disorders for women in rural areas (since many of the emigrants were young men who thus left a huge workload for those left behind to bear. See Uimonen, *Hermostumisen aikakausi*, 104

38 For another example of a troubled young girl, see ‘Hänen tarpeensa’, *Terveydenhoitolehti*, 1900, 8, 61–2.

39 ReijoWaara, ‘Tytöstä naiseksi kehittyessä’, 5.

40 Nature, work and the countryside (e.g. taking care of animals) were still being regarded as important ways to heal and ‘morally’ remediate asocial children even in the mid-twentieth century. See Vehkalahti, ‘Kasvatuslaitos modernin kansalaisuuden rakentajana’, 395.

41 Markkola, ‘Negotiating Family, Education, and Labor’.

42 Saara Tuomaala, ‘Kamppailu yhteisestä koulusta ja oppivelvollisuudesta’, 95–100.

43 Rahikainen, ‘School, Child Labour and Rationalization’; Rahikainen, ‘Lasten työnteko ja lapsityövoiman kysyntä.’

44 Halmesvirta, *Vaivojensa vangit*, 145–46.

45 Turo-Kimmo Lehtonen, ‘Bakteerit ja henkisten ruttotautien siemenet. Puhdas elämä suomalaisessa terveysvalistuksessa 1890-luvulla’, in Timo Joutsivuo and Heikki Mikkeli, eds, *Terveyden lähteillä. Länsimaisten terveyskäsitysten kulttuurihistoriaa* (Helsinki: SKS, 1995), 220–22.

46 Vihtori Peltonen, ‘Elonilmauksia ihmisen elämässä’, *Suomen Terveydenhoitolehti*, 1894, 6, 140.

47 See, for example, Konrad Relander, ‘Työn siunaus’, *Suomen Terveydenhoitolehti*, 1892, 4, 33–4, and *Konrad* Relander, ‘Neuvoja kansakoulunopettajille ja muillekin varteenotettaviksi’, *Suomen Terveydenhoitolehti*, 1890, 2, 42–3. To help primary school teachers keep their nerves in balance, ReijoWaara suggested healthy and joyful outdoor activities, such as gardening.

48 Halmesvirta, ‘Despair in Finnish’, 150; Roy Porter, *The Greatest Benefit to Mankind* (London: Fontana Press, 1999), 221–47.

49 See Sally Shuttleworth, ‘Art of Medicine. Victorian Visions of Child Development’, *Lancet*, 2012, 379 (January 21), 212–13.

50 ‘Ääniä kansakoulun työmaalta. Kansakoulu käytännöllisemmäksi’, *Kansakoulun lehti*: *kasvatusopillinen aikakauskirja kodille ja koululle*, 1905, 23 (16–17), 611–12.

51 A. Hj. Ilmoni, ‘Koulunuorison liikarasituksesta’, *Suomen Terveydenhoitolehti,* 1897, 9, 70–1.

52 Konrad Relander, *Koululääkärikysymyksen vaiheet Suomessa* (Helsinki: Suomalaisen Kirjallisuuden Seura, 1896).

53 Relander, *Koululääkärikysymyksen vaiheet*, 1896, 8, 13–5.

54 Konrad Relander, ‘Hermoheikkous ja lepopäivä’, *Suomen terveydenhoitolehti*, 1890, 2, 73–74.

55 Like ReijoWaara, Ruotsalainen was one of the editors in the *Finnish Health Care Journal*, and between 1919 and 1931, he was chief editor.

56 Armas Ruotsalainen, ‘Taipumus hermotauteihin lapsuuden iässä ja sen hoito’, *Suomen Terveydenhoitolehti,* 1916, 28, 165–68.

57 Ibid., 166.

58 See, for example, Onni Granholm, *Kärlek. Normal och abnormal form* (Helsingfors: Söderström & Co, 1900), 88–89.

59 Ruotsalainen, ‘Taipumus hermotauteihin’.

60 For instance, Anon., ‘Hermostuneisuus ja kasvatus’, *THL* 1/1902, 14–6.

61 Letter to ReijoWaara 31 January 1916.

62 ‘Huolestunut’ (1072), *Suomen Terveydenhoitolehti*, 1916, 28, 46.

63 ‘Huolestunut äiti’ (1047), *Suomen Terveydenhoitolehti*, 1911, 23, 31.

64 Ruotsalainen, ‘Taipumus hermotauteihin’, 168

65 Onni Granholm, ‘De psykiska förhållandena vid späda barns vård’, *Finska läkaresällskapets handlingar*, 1908, L, 167.

66 Ibid., 169–71.

67 Anon., ‘Miten tullaan oikeiksi kasvattajiksi’, *Aikakauslehti lasten wanhemmille ja kaswattajille* 1 January 1900.

68 Tarja Kakko, *Kurissa ja Herran nuhteessa.* Luterilainen kasvatusaate 1900-luvun vaihteen Suomessa (Turku: Turun yliopisto, 2005), 90–100.

69 Markku Aukia, *Lapset olkoon ihmisiksi. Suomen talonpoikien hiljainen kasvatus. Kansatieteellinen tutkimus* (Turku: University of Turku, 2009), 207–209. For example, a child who wept and cried uncontrollably when punished could be called *sydämikkö* (‘sydän’ means heart), and this child usually was not punished. This could be considered as a folk diagnosis of a child’s personality. There was also a saying that ‘you cannot whip a speechless child’.

70 For instance, ‘Hänen tarpeensa’, 61–62.

71 Birgitta Tommola, *Yliluonnolliset olennot lapsenpelotuksina* (Helsinki: SKS, 1955).

72 E.L., ‘Älkää peloitelko lapsia!’, *Suomen Terveydenhoitolehti*, 1913, 25, 193.

73 Halmesvirta, *Vaivojensa vangit*, 158–60.

74 For example, James Crichton Browne, Benno Huisinga, Lord Shaftesbury, and several Swedish and Danish experts.

75 Anon. ‘Liiallisesta henkisestä työstä kouluissa ja heikkohermoisuudesta’, in *Suomen Terveydenhoitolehti*, 1895, 7, 184–85.

76 Bakker, ‘Before Ritalin’, 384.

77 Halmesvirta, *Vaivojensa vangit,* 156–64.

78 For example, Rudolf Virchow in 1848.

79 Provincial medical officers were state-employed officials with a high status and clearly defined legal obligations that were mainly focused on public health. See Mikko Jauho, ‘From the Local to the Global, from the Environment to the Individual. Epidemiological Knowledge Production and Changing Notions of Public Health’, in Esa Ruuskanen and Heini Hakosalo, eds, *In Pursuit of Healthy Environment. Historical Cases on the Environment-Health Nexus* (London and New York: Routledge, 2021), 201.

80 The study was focused on socio-economic determinants of health, and it was exceptionally detailed, even ‘ethnographic’, in its scope. Besides taking into account many kinds of social and environmental issues, it examined peoples’ behaviour and way of life in such detail that ‘he succeeded in finding out that peasant women wore no underwear, except those few who had earlier emigrated to America’. See Eero Lahelma, Antti Karisto and Ossi Rahkonen, ‘Analysing Inequalities. The Tradition of Socioeconomic Public Health Research in Finland’, *European Journal of Public Health* 6(2) (1996), 89; see also Jauho, ‘From the local to the global’, 203–204.

81 For instance, letters by ‘Nuori’, ‘Nuori mies’, and ‘17-vuotias’. All date from 1908.

82 Letter from ‘Nuori’ to Dr ReijoWaara (1908)

83 Johannisson, *Medicinens öga. Sjukdom, medicin och samhälle: historiska erfarenheter* (Stockholm: Norstedts, 1990), 115; Doris Kauffmann, ‘Neurasthenia in Wilhelmine Germany: Culture, Sexuality, and the Demands of Nature’, in Gijswijt-Hofstra and Porter, eds, *Cultures of Neurasthenia*, 161–62; Thomas W. Laquer, *Solitary Sex: A Cultural History of Masturbation* (New York: Zone Books, 2003), 37–41. Especially after Swiss doctor Simon-André Tissot published his *L’Onanisme* (1750), masturbation was increasingly treated as a particular kind of disease with a range of symptoms and more or less severe after-effects.

84 Letter from ‘Huolestunut 17-vuotias’ (1908).

85 For instance, a letter from ‘Fix’ (31 March 1908).

86 See Laquer, ‘Solitary Sex’, 194–96; Arja-Liisa Räisänen, *Onnellisen avioliiton ehdot. Sukupuolijärjestelmän muodostumisprosessi suomalaisissa avioliitto- ja seksuaalivalistusoppaissa 1865–1920* (Helsinki: SHS, 1995), 187–88.

87 A positive exception among the vast majority of cases was a 15-year-old boy, who asked if it would be reasonable to ‘try out copulation’ if it would help him quit masturbating. It appeared the boy got the idea from some ‘foreign magazine’. According to ReijoWaara, however, this advice from the foreign doctor was ‘absolutely harmful’. See ‘15-vuotias’ (560), *Suomen Terveydenhoitolehti*, 1908, 20, 134

88 Letter from ‘Patiently waiting 16 yr-old’ (*16 v. Kärsivä ja odottava*), 1908.

89 Letter from ‘Artturi’ (29 January 1908).

90 Sylvanus Stall, *Mitä nuoren miehen tulee tietää: “nuorukaisen ylpeys on hänen voimansa”.* Tahvo Partio, transl. (Helsinki: Yrjö Weilin, 1905). Stall (1845–1917) was a Lutheran pastor from the USA who had written a sex education book called *What a Young Boy Ought to Know* (1897).

91 Letter from ‘18-year-old’ (26 October 1908).

92 For instance, a diet with ‘lots of berries and dairy products’ and plenty of outdoor activities (e.g. swimming, skiing and ‘walking barefoot’). ‘Toivoton 17-vuotias’ (563), *Suomen Terveydenhoitolehti*, 1908, 20, 150.

93 She attended sixth grade in a mixed middle school which at this time meant the first grade in a high school.

94 This was a book written by the American doctor, social reformer and author of self-help books for women, Mary Wood-Allen—translated into Finnish as ‘Mitä tytön tulee tietää’ (1908).

95 It is impossible to verify the sex of this ‘friend’, but the phrasing implies these were sexual feelings for another female.

96 She writes that she had solved the problem of masturbation by ‘tying her hands on the back of her head during the night’.

97 The tendency towards detailed self-observation and ‘listening out’ for any worrying physical signs was a very common feature in these kinds of letter.

98 As rented farms, crofts were usually situated far from the village centre. Crofter families paid their rent in cash or kind—as labour or goods (e.g. wool yarn, berries or agricultural produce).

99 Markkola, ‘Negotiating Family, Education, and Labor’, 165. In 1900, around 87 per cent of Finns lived in rural areas and agriculture was the main source of income for most of the Finnish population, however, less than a half earned their living by independent farming.

100 In these kinds of circumstances, industrial labour could represent a much-needed positive alternative to poverty and destitution. See ibid., 169.

101 Maarit Leskelä-Kärki, ‘Constructing Sisterly Relations in Epistolary Practices: The Writing Krohn Sisters (1890–1950)’, *Nordic Journal of Feminist and Gender Research*, 2007, 15, 21–34.

102 Lehtonen, ‘Bakteerit ja henkisten ruttotautien siemenet’, 220–26.

103 See, for example, Anne Harrington, *Mind Fixers. Psychiatry’s Troubled Search for the Biology of Mental Illness* (New York and London: W.W. Norton & Company, 2019), 74–81. Alice Smuts, *Science in the Service of Children* (New Haven and London: Yale University Press, 2006).

104 See, for example, Sophy Bergenheim, ‘Helping Troubled Children and Cultivating the Race: The Mental Hygienic Guidance Centres of the Public Health Association of Swedish Finland, 1930s-1950s’, *Social History of Medicine*, 2020, 33, 1282–305.

105 Mika Ojakangas, ‘Mentaalihygienia ja lapsuus. Lapsen sopeutuminen onnellisuuden ja hyvinvoinnin ehtona 1920–40-lukujen Suomessa’, in Joutsivuo and Mikkeli, eds, *Terveyden lähteillä,* 291–313; Myllykangas and Parhi, ‘Unjustified Emotions’, 493–99; Oona Ilmolahti, *Eheys ja ennakkoluulo* (Helsinki: THPTS, 2017), 379–85; Mirja Satka and Timo Harrikari, ‘The Present Finnish Formation of Child Welfare and History’, *British Journal of Social Work*, 2008, 38, 654–55.

106 Cf. Anna Christina Rose, ‘Between Psychology and Pedagogy. “Moral Orthopedics” and Case Studies of Children in Fin-de-Siècle French Medicine’, *History of Psychology*, 2001, 14, 26–52.

107 Halmesvirta, ‘Despair in Finnish’, 139–41.

108 As Mikko Jauho has recently pointed out, the main paradigm guiding public health policy during this era of ‘sanitary statistics’ (both in Finland and internationally) was *miasma theory*, that is, poisoning from contaminated air, soil or water. Preventive measures were thus characteristically social and environmental—for example, improving sanitation and building better houses. Jauho, ‘From the Local to the Global’, 196–98.

109 Lahelma, Karisto and Rahkonen, ‘Analysing Inequalities’. Upheavals among the working class framed the problems of childhood in a new way—the young (boys especially) were now seen as being susceptible to communist ideas. See also Lauri Julkunen, ‘Yrjö Karilas nuorisokasvattajana ja kristillisen poikatyön kasvatustavoitteiden määrittelijänä 1920- ja 1930-luvulla’, *Kasvatus & Aika*, 2014, 8, 61–78.

110 ‘For a long time my head has been very sensitive to touch, especially the middle of my head. I’ve been trying to fix this lately by reading as much as I can and reading a lot of wisdom.’

